# Coastal urbanisation affects microbial communities on a dominant marine holobiont

**DOI:** 10.1038/s41522-017-0044-z

**Published:** 2018-01-17

**Authors:** Ezequiel M. Marzinelli, Zhiguang Qiu, Katherine A. Dafforn, Emma L. Johnston, Peter D. Steinberg, Mariana Mayer-Pinto

**Affiliations:** 1 Centre for Marine Bio-Innovation, School of Biological, Earth and Environmental Sciences, The University of New South Wales, Sydney, NSW 2052 Australia; 2 Sydney Institute of Marine Science, 19 Chowder Bay Rd, Mosman, NSW 2088 Australia; 3 Singapore Centre for Environmental Life Sciences Engineering, Nanyang Technological University, 60 Nanyang Drive, SBS-01N-27, Singapore, 637551 Singapore; 4 Applied Marine and Estuarine Ecology Lab & Evolution and Ecology Research Centre, School of Biological, Earth and Environmental Sciences, The University of New South Wales, Sydney, NSW 2052 Australia

## Abstract

Host-associated microbial communities play a fundamental role in the life of eukaryotic hosts. It is increasingly argued that hosts and their microbiota must be studied together as 'holobionts' to better understand the effects of environmental stressors on host functioning. Disruptions of host–microbiota interactions by environmental stressors can negatively affect host performance and survival. Substantial ecological impacts are likely when the affected hosts are habitat-forming species (e.g., trees, kelps) that underpin local biodiversity. In marine systems, coastal urbanisation via the addition of artificial structures is a major source of stress to habitat formers, but its effect on their associated microbial communities is unknown. We characterised kelp-associated microbial communities in two of the most common and abundant artificial structures in Sydney Harbour—pier-pilings and seawalls—and in neighbouring natural rocky reefs. The kelp *Ecklonia radiata* is the dominant habitat-forming species along 8000 km of the temperate Australian coast. Kelp-associated microbial communities on pilings differed significantly from those on seawalls and natural rocky reefs, possibly due to differences in abiotic (e.g., shade) and biotic (e.g., grazing) factors between habitats. Many bacteria that were more abundant on kelp on pilings belonged to taxa often associated with macroalgal diseases, including tissue bleaching in *Ecklonia*. There were, however, no differences in kelp photosynthetic capacity between habitats. The observed differences in microbial communities may have negative effects on the host by promoting fouling by macroorganisms or by causing and spreading disease over time. This study demonstrates that urbanisation can alter the microbiota of key habitat-forming species with potential ecological consequences.

## Microbial–host interaction: Holobionts and harbours

Artificial structures in the marine coastal environment, such as piers and seawalls can alter microbial interactions with seaweed hosts. Researchers in Australia, led by Ezequiel Marzinelli at the University of New South Wales, studied the microbial communities growing on kelps in Sydney Harbour. They compared this altered environment with neighbouring natural rocky reefs. The work illustrates the “holobiont” concept, which treats communities of microbes and hosts as holistic entities. Significant findings included an increase in microbes associated with algal diseases in pier piling structures. This study shows that urbanisation of the coastal environment can have ecological consequences on major habitat-forming species such as kelp. The researchers argue that the combination of abiotic factors, such as shade, and biotic factors, such as altered grazing patterns, might be involved.

## Introduction

Increasing evidence from a wide range of systems, such as human biology,^1^ plant/soil interactions,^2^ reef building corals^3^ and seaweeds^4^ suggest that host-associated microbial communities are critically important for the development,^5^ health^6^ and defence^1^ of eukaryotic hosts. It is increasingly apparent that in order to understand the factors that influence the functioning of eukaryotes, it is important to study them as 'holobionts', that is, hosts and their associated microbial communities as a whole.^3,4,6,7^

Many of the world’s ecosystems are dominated by habitat-forming holobionts. These organisms, such as trees on land, or reef-building corals and macroalgal forests in the oceans, facilitate other organisms by modifying the surrounding environment, supporting diverse and productive communities.^8–10^ Impacts on the interaction between habitat-forming hosts and their associated microbial communities are critical because such effects can cascade throughout an entire ecosystem. In marine temperate systems on rocky shores, kelps (macroalgae) underpin biodiversity, and crucial coastal ecosystem functions and services.^10,11^ Key habitat-forming kelps are, however, increasingly under threat from multiple human stressors such as habitat modification and climate change, which are leading to declines of kelp forests around the world.^12–16^

Urban coastal systems in particular are examples of highly modified ecosystems, with a diversity of built infrastructure transforming shorelines worldwide in what has been coined 'ocean sprawl'.^17,18^ Indeed, more than 50% of the coastline around numerous cities in Europe, USA, Australia and Asia has been modified by the addition of artificial structures built for defence and recreational purposes, such as breakwaters, seawalls and marinas.^19,20^ Coastal development and marine infrastructure is likely to increase in the future with the rapid increase in the human population inhabiting coastal areas, and in response to predicted increases in sea-level rise and the frequency and severity of storms.^20^

Artificial structures typically replace and/or fragment natural habitats, and are generally built with materials and physical characteristics that do not resemble the natural habitats they replace.^20,21^ These structures can alter abiotic factors, such as light availability and water-flow, as well as ecological interactions, such as grazing pressure and fouling, having direct and indirect effects on associated biodiversity.^22–25^ These environmental and ecological changes are likely to have the strongest impacts where the affected organisms are habitat-forming or 'foundation' species such as kelps^26^ because they can have disproportionate effects on biodiversity and ecosystem functioning.^27^

While we have substantial understanding as to how artificial structures modify coastal environments and support different macrofaunal communities relative to natural habitats,^17,20^ the consequences of habitat modification for microbial communities remain largely overlooked. In particular, our understanding of how coastal urbanisation affects microbial communities associated with marine habitat-forming hosts is essentially non-existent.

To our knowledge, only one study so far has investigated microbial communities associated directly with the substratum (biofilms) on natural (rocky reefs) and artificial (seawalls) habitats in coastal systems.^28^ The structure of these intertidal bacterial communities differed between seawalls and rocky shores, suggesting that built infrastructure not only affects macroorganisms, but also initial colonisation by biofilms on their surfaces. Given that biofilm structure and composition can influence colonisation of macroorganisms,^29–32^ this finding suggests that artificial structures may indirectly influence the prevalence and abundance of some macroorganisms via changes in the biofilms.

We examined the structure of kelp-associated microbial communities on two of the most common and abundant artificial structures in Sydney Harbour—pier-pilings, which are typically built using wood or concrete and replace soft-sediment habitats and, in some cases, modify natural rocky reefs, and seawalls, which are typically built using sandstone or concrete and replace or fragment natural rocky reefs^33^—and compared them with natural rocky reef communities. The kelp *Ecklonia radiata* (hereafter *Ecklonia*) is the dominant habitat-forming species on over 8000 km of Australian temperate reefs,^34,35^ including Sydney Harbour.^36^ Sydney Harbour is one of the largest urbanised harbours in the world with >50% of the shoreline modified by seawalls and pier-pilings supporting more than 40 functioning marinas, as well as private jetties and swim-nets.^37,38^ Although these structures support different biodiversity from adjacent natural rocky reefs, they still provide habitat for *Ecklonia*. However, kelp are negatively affected by such structures, where they experience lower light availabily and greater fouling.^24,39–41^

Microbial communities associated with surfaces of kelp growing on pilings, seawalls and natural rocky reefs were characterised using 16 S rRNA gene tag sequencing, and the photosynthetic efficiency of these kelp was also quantified. We predicted that the microbial communities associated with kelp on artificial structures would differ from those on kelp in adjacent natural rocky reefs due to differences in environmental conditions between the types of habitat, and that these differences would also affect the host such that photosynthetic efficiency of kelp would be lower on artificial structures than on natural reefs.

## Results

**Fig. 1:**
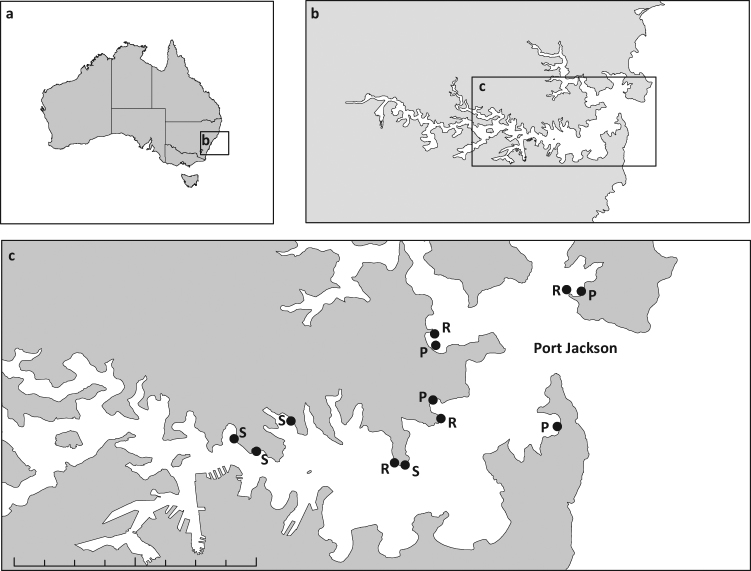
Location of study sites. Map of Sydney Harbour (**b**, **c**), Australia (**a**), showing the locations from where kelp-associated microbiomes were sampled. R rocky reefs, S seawalls, P pier-pilings. Scale bar: 6 km

**Fig. 2:**
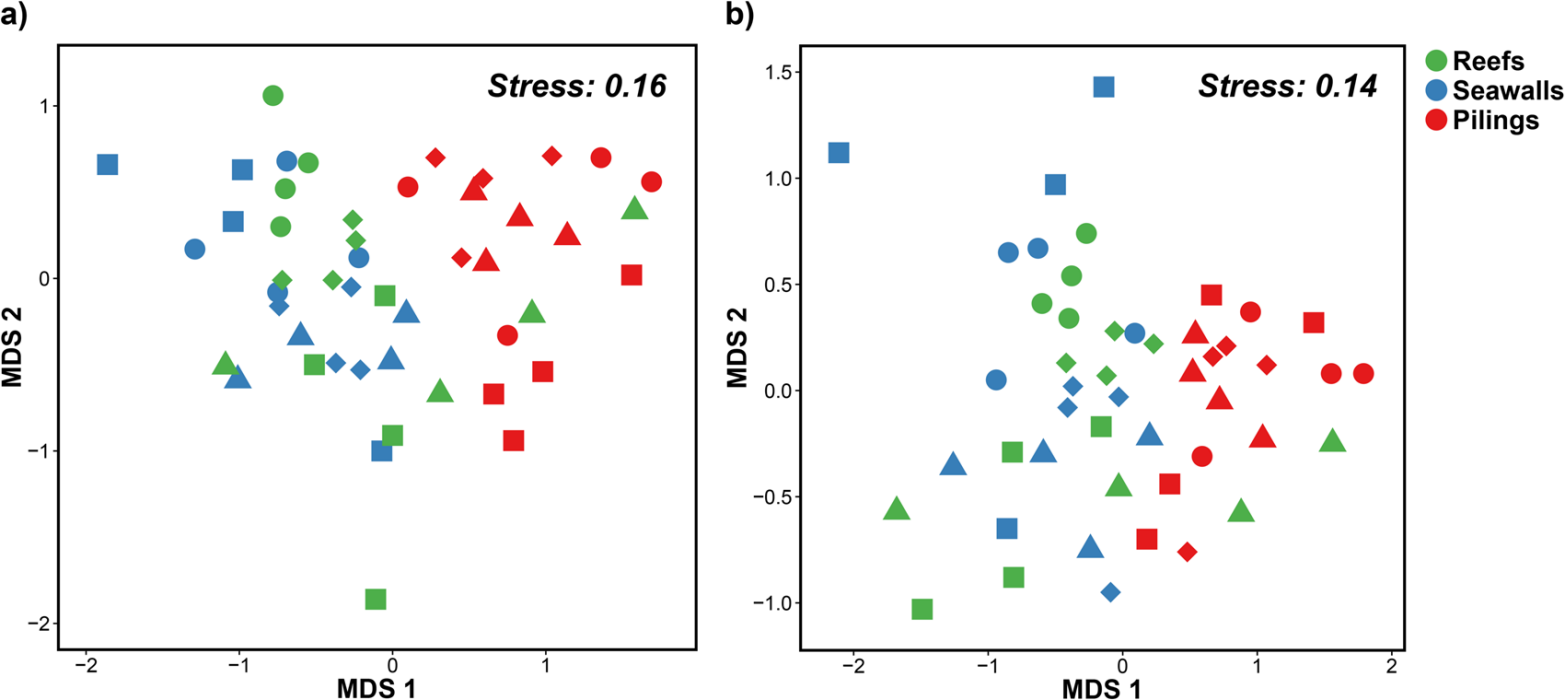
Kelp-associated microbial communities in the three habitat types. nMDS based on the **a** Bray–Curtis or **b** Jaccard measure on square-root transformed relative abundances of OTUs on kelp in the three habitat types sampled: natural rocky reefs (green symbols), seawalls (blue symbols) and pilings (red symbols). Different shapes represent the different sites sampled in each habitat type

*Ecklonia radiata* samples were collected at each of four, mostly spatially interspersed, wooden pier-pilings, sandstone seawalls or natural sandstone reefs sites in Sydney Harbour, Australia, totalling 12 sites (Fig. 1). The relative abundance and composition of microbial communities associated with kelp on pilings differed significantly from those on seawalls and natural rocky reefs, which did not differ from each other (Fig. 2, Table 1). There were no differences in multivariate dispersion, which measures variability of the microbial communities among habitat types (PERMDISP analysis: Bray–Curtis, *F*_2,36_ _=_ 0.81, *p* = 0.55; Jaccard, *F*_2,36_ _=_ 0.32, *p* = 0.28).

**Table 1:** PERMANOVA analyses based on (a) Bray–Curtis and (b) Jaccard measures of square-root transformed relative abundances of OTUs associated to the surfaces of kelp in each habitat type (Ha, fixed; rocky reef ‘R’, seawalls ‘S’ and pilings ‘P’) and site (Si, random, nested in habitat, with four levels each)

		(a) Bray–Curtis			(b) Jaccard		
Source	d*f*	MS	Pseudo-*F*	*P*	MS	Pseudo-*F*	*P*
Ha	2	6946.2	2.1709	**0.008**	6527.9	1.6339	**0.004**
Si(Ha)	9	3199.7	2.0146	**<0.001**	3995.2	1.3491	**<0.001**
Res	36	1588.3			2961.3		
Total	47						

Pairwise tests: R ≠ P, S ≠ P, S = R (for Bray–Curtis and Jaccard)

**Fig. 3:**
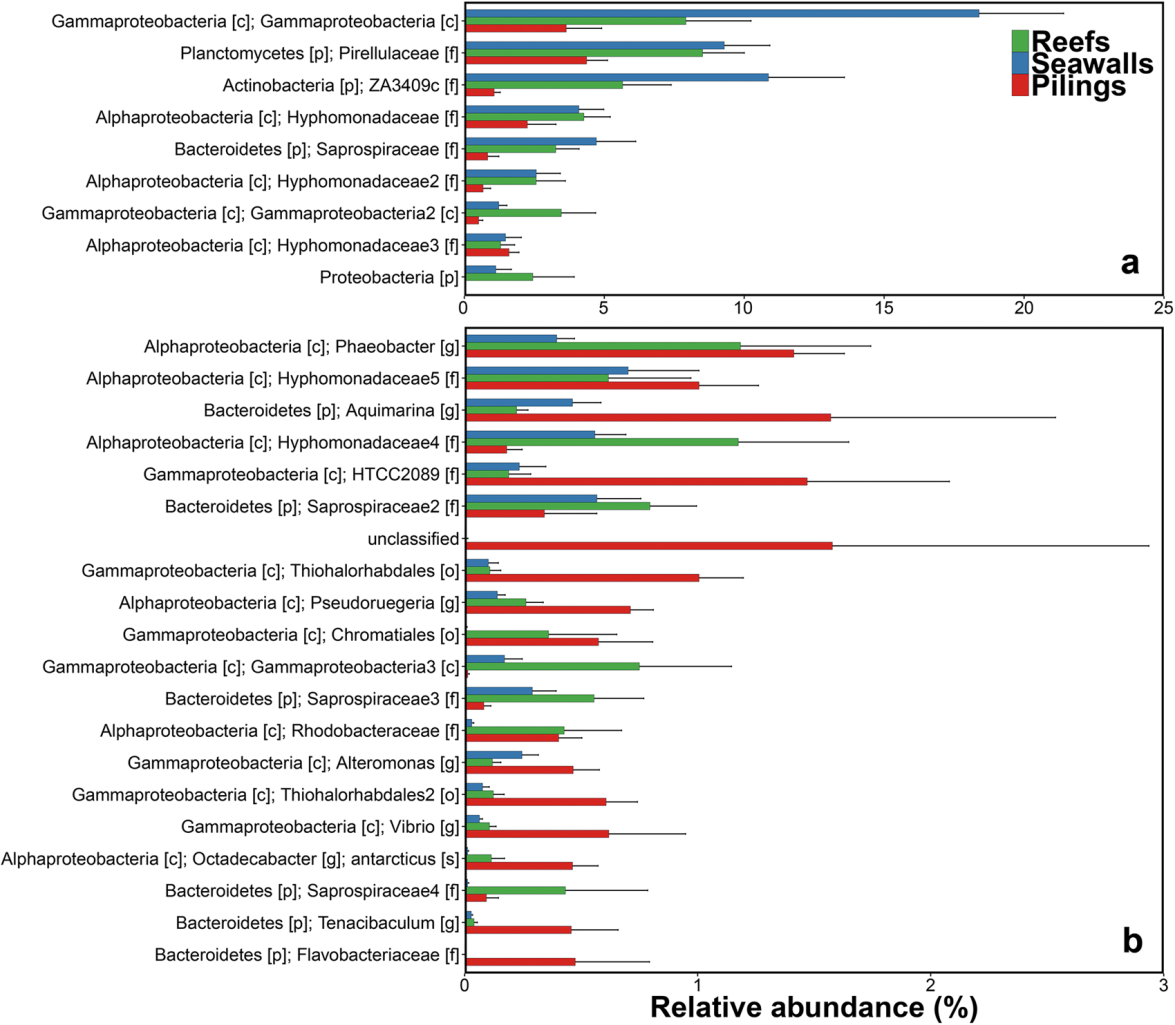
Differences in abundances of the dominant OTUs between habitat types. Mean relative abundances (+s.e.m., *n* = 16) of OTUs found to differ strongly between the three habitat types sampled: natural rocky reefs (green bars), seawalls (blue bars) and pilings (red bars) (p phylum, c class, o order, f family, g genus, s species). **a** OTUs with relative abudances greater than 4%; **b** OTUs with relative abundances smaller than 4%

Multivariate generalised linear models (GLMs) identified 27 OTUs whose relative abundances differed significantly among the different habitats, and for which the size of the effect was greater than twice the standard error (Fig. 3, Table S2). The relative abundances of over a third of these OTUs were found to differ between kelp on natural rocky reefs and kelp on pier pilings. An OTU assigned to the phylum Proteobacteria, several OTUs in the class Gammaproteobacteria and some in the family Saprospiraceae were significantly more abundant (~50%) on kelp on reefs than on seawalls or pilings. Generally, abundances of these OTUs were higher on kelp on seawalls than on pilings (Fig. 3).

In contrast, OTUs assigned to the genera *Aquimarina*, *Alteromonas* and *Vibrio*, as well as those in the family *Flavobacteriaceae* and some OTUs in the class Gammaproteobacteria (order Thiohalorhabdales and family *HTCC2089*) were significantly more abundant (>50%) on kelp on pilings than on kelp on seawalls or reefs (Fig. 3). One OTU assigned to the class Gammaproteobacteria and another to the phylum Actinobacteria were 30–50% more abundant on kelp on seawalls than on reefs, which in turn were more abundant than on kelp on pilings (Fig. 3).

Kelp maximum photosynthetic yield did not differ among the three habitats (mean ± s.e.m., *n* = 32, pilings: 703.3 ± 12.7, reefs: 675.9 ± 13.7, seawalls: 697.8 ± 22.3; *χ*^2^ = 0.68, d*f* = 2, *p* = 0.71).

## Discussion

Given the fundamental role that microorganisms play on the functioning of eukaryotic hosts, investigating responses of both hosts and associated microbial communities is crucial to understanding the ecological consequences of urbanisation. This information is largely lacking for habitat-forming species, such as macroalgae in coastal systems. We found that the microbial communities associated with the surfaces of the kelp *Ecklonia radiata* on pilings were consistently different from those on kelp on seawalls or rocky reefs, and some specific OTUs also differed between kelp on seawalls than rocky reefs. Interestingly, many of the bacteria that were more abundant on kelp on pilings belong to taxa often associated with macroalgal diseases, including tissue bleaching in *Ecklonia*, which is common and widespread across the kelp’s latitudinal distribution in Australia.^42^ There were, however, no differences in the maximum photosynthetic capacity of kelp between habitats, suggesting that differences in environmental conditions and associated differences in microbiomes between these habitats may not affect host functioning directly, although there may be effects on other functions of the host not quantified here, such as respiration fluxes.^43^

The observed differences in the kelp-associated microbial communities may be due to several environmental factors. For example, kelp on pilings typically receive significantly lower levels of light than those on reefs or seawalls because the pier supported by the pilings shade them.^22,24^ This factor has been shown to cause differences in fouling by macroorganisms between these habitats and may thus also influence microorganisms. In addition, the material onto which the algae are attached may also be a factor influencing the observed differences,^28,44^ although the microbial communities were sampled from secondary blades at mid-thallus, which is not directly in contact with the substratum. Kelp on pilings can also have higher contaminant loads relative to kelp on natural reefs,^45^ which can affect microbial community structure.^46^ Potential differences in the microbial communities in the water column between these habitats may lead to differences in the surface-associated microbiota on kelp. We did not sample microbial communities in the water because previous studies directly comparing host-associated microbial communities with those in the adjacent water column have found no relationship between them.^47–49^ Host-associated communities and those in the water are typically very different, potentially because the host may act as a selective 'filter, selecting microorganisms which are usually very rare in the water, but that become abundant once they establish on host surfaces.^47^

The kelp-associated microbial communities may be also affected by differences in ecological processes and/or interactions occurring in each type of habitat. For instance, kelp on pilings are significantly more fouled by encrusting and erect bryozoans and hydroids, most of which are non-indigenous, than kelp on adjacent natural reefs.^40,41^ They also experience less disturbance/grazing by the canopy-dwelling sea urchin *Holopneustes purpurascens*, which is found in much lower abundances on pilings than on natural reefs.^24^ Thus, greater fouling and/or lower disturbance of the kelp surface could be driving the observed differences in associated microbial communities.

Several bacterial taxa that were found to be more abundant on kelp on pilings are also associated with putative algal diseases. These bacterial taxa include the genera *Aquimarina*, *Alteromonas* and *Vibrio*, and the family Flavobacteriaceae.^42,50^ Furthermore, some of these taxa were the causative agents of disease as shown in inoculation experiments.^51^ Many bacteria in the genus *Aquimarina* can degrade polysaccharides of algal surfaces^52^ and *Alteronomas* spp. have been associated to lesions and bleaching of algal tissue in other hosts,^51,53^ and both are consistently found on bleached *Ecklonia.*^42^ We do not yet know if these particular bacterial taxa cause disease in *Ecklonia*—although this has been shown for another co-occurring macroalga^51^—but this finding suggests the algae on pilings may be at greater risk from disease than those in other habitats. Artificial structures such as those studied here are becoming more abundant worldwide.^18^ Given that bacteria associated with diseases of habitat-formers were highly enriched in these habitats, further addition of these structures may result in an overall increase of putative pathogens, potentially facilitating the spread of diseases to adjacent natural habitats.

In contrast to other studies that have found strong links between changes in the associated microbial community and the condition and performance of the host, e.g., refs. ^42,54,55^ we did not find differences in the photosynthetic capacity of kelp among the habitats. However, changes in the microbiota may negatively affect other processes not measured here, such as day/night respiration fluxes, which can lead for example to hypoxic zones, thus negatively affecting the kelp host.^43^ Nevertheless, despite the apparent absence of direct effects, differences in the associated microbial communities may affect host functioning indirectly, via changes in ecological interactions. It is possible that differences in amounts of fouling by bryozoans and hydroids may not be a cause of the observed differences in the kelp microbiome between habitats, but a consequence. For instance, greater shading on pilings may directly affect kelp microbiota, which, in turn, may make the algae more susceptible to the colonisation of fouling organisms.^39^ The dominant epibiont on kelp on pilings in Sydney Harbour is the non-indigenous encrusting bryozoan *Membranipora membranacea*^40^ which recruits in much higher numbers and grows up to four times faster in this habitat than in natural reefs, and can cover over 50% of the kelp thallus.^23,39^ Several studies have shown that larval settlement of bryozoans can be influenced by the microbiome on the surfaces onto which they settle and colonise,^30,56^ and may thus be the mechanism behind the higher abundance of *M. membranacea* on kelp on pilings.^23,39^ Greater fouling can eventually lead to lower photosynthetic capacity and increased tissue loss due to increased drag, fragmentation and consumption by predators that target the fouling organisms, indirectly damaging the fouled kelp tissue.^57,58^ If this model is true, it implicates an interesting link between artificial structures, microbiomes and biological invasion of fouling organisms. Further experiments would help unveil the explanatory models proposed here.

The holobiont paradigm is transforming our understanding of biomedical science, including new approaches to disease management,^6,59^ and there is increasing evidence that this new integrative paradigm may be similarly transformative in understanding environmental systems.^60,61^ This is particularly critical in the context of human impacts since stressful environments may cause the loss of microorganisms with critical functions for host performance.^7,62,63^ We know almost nothing about how urbanisation affects host-associated microbiomes and how this can influence host functioning and resilience to stress in marine coastal systems. Our study shows that urbanisation can have further potential impacts on ecological systems by altering the microbial communities associated to key habitat-forming species. Urban structures may act as havens where putative pathogens can thrive and may thus facilitate the spread of diseases to habitat-forming hosts in adjacent natural habitats.

## Material and Methods

### Sample collection

*Ecklonia radiata* samples were collected at each of four wooden pier-pilings, sandstone seawalls or natural sandstone reefs sites in Sydney Harbour, Australia, totalling 12 sites (Fig. 1). Sites were sampled in a haphazard order, with approximately half of the sites for each habitat type sampled on 30 March 2014, and the remaining sites on 5 April 2014. The majority of the sites per habitat type were spatially interspersed, although there was some spatial structure for seawall sites, which occur predominantly west of the Harbour (Fig. 1). The 'body' or thallus of adult *Ecklonia* individuals consists of a holdfast, which serves as an anchoring point to the substratum, a stipe and the blades. The stipe supports a primary blade from which secondary blades protrude. At each site, secondary blades from the middle section of the thallus (~10 cm above the stipe) of seven kelp ~2 m apart were sampled underwater on snorkel at 1–2 m depth. Some kelp on pilings were fouled by invertebrates (<20% of the blades), mainly encrusting bryozoans. Areas of the tissue that were not fouled were specifically targeted to ensure sampling of only the microbiome associated to kelp tissue. Tissue samples were placed individually in press-sealed bags and brought to the surface where they were rinsed with filtered seawater to remove unattached microorganisms. The microbial communities on the middle section of each secondary blade were then sampled with sterile cotton tips, which were used to gently swab approximately 20 cm^2^ of each algal surface for 30 s, as in previous research in our group.^42,62^ Each swab was then immediately aseptically transferred into a cryogenic tube and snap-frozen in liquid nitrogen contained in a dry shipper and then stored at −80 °C at the University of New South Wales. A subset of *n* = 4 samples per site were used for sequencing (below).

At the time of sampling, an adjacent section of each tissue sample (*n* = 7 per site) was dark-adapted in situ for 15 min using the dark-adapting accessory clips provided by the manufacturer (‘Leaf Clip’ DLC-8, Walz, Germany) and the maximum photosynthetic quantum yield (i.e., the maximal light utilisation efficiency in the dark; *Fv*/*Fm*) was quantified using a Pulse Amplitude Modulated fluorometer (Diving-PAM; Walz, Germany).^42^ Photosynthetic measures were obtained between 10 am and 3 pm.

### DNA extraction and sequencing

Microbial DNA from a subset of the samples (*n* = 4 per site; *N* = 48) was extracted from each cotton tip using a Powersoil DNA Isolation Kit (MO Bio Inc. Carlsbad CA, USA) following the protocol provided by the manufacturer. The purity and quantity of DNA extracts were determined by agarose gel electrophoresis and spectrophotometric NanoDrop-1000. DNA were stored at −20 °C for further use.

The extracted DNA samples were amplified with PCR by using 16 S rDNA primers 515 F (5′-GTGYCAGCMGCCGCGGTAA-3′) and 806 R (5′-GGACTACNVGGGTWTCTAAT-3′) that contain the conserved region V4 of the bacterial 16 S rRNA gene, as per our previous work on kelp microbiomes.^42^ The amplicons were purified with a gel recovery method (Zymo DNA-5 Clean Concentrator) before they were sent to the Ramaciotti Centre for Genomics (UNSW, Australia) for sequencing using the Illumina MiSeq 2000 platform.

### 16 S rRNA gene processing and quality filtering

Raw data acquired from sequencing were quality filtered, standardised, classified and then clustered into operational taxonomic units (OTUs) using the sequencing analysis software Mothur.^64^ Briefly, sequences including forward and reverse reads (251 bps per read) were firstly combined into contigs. Sequences that contained N bases or had >8 homopolymers were filtered out. Remaining sequences were aligned referring to the Silva 16 S rRNA gene database^65^ and sequences that did not align were excluded. Sequences aligned were pre-clustered (diffs = 2) and checked for chimeras using UCHIME.^66^ Singleton and doubleton sequence reads were removed from the data set to reduce further noises caused by Illumina sequencing error.^67^ Remaining sequence counts were rarefied to 44,225 reads per sample to account for differences in sequencing depth. Sequences were then taxonomically classified according to the Silva 16 S rRNA gene database with 60% cut-off confidence and clustered into OTUs at a minimum of 97% taxonomic identity. Rarefaction curves of the processed sequences were generated to estimate sampling efficiency (Supplementary Fig. S1). High-quality sequences selected from the raw data set resulted in 13,978 OTUs clustered at 97% similarity. In order to focus analyses on the abundant OTUs and reduce the effect of potentially spurious OTUs, those that contribute to less than 0.01% of relative abundance were removed from the data set, which resulted in 475 OTUs that were used for further analyses.

### Statistical analysis

The OTU table generated above was analysed using permutational multivariate analysis of variance (PERMANOVA)^68^ in the PERMANOVA+ add-on in PRIMER v6 (PRIMER-E, UK) to compare microbial communities on kelps in different habitats. ‘Habitat’ was a fixed factor with three levels (natural rocky reefs, seawalls and pilings) and ‘Site’ was a random factor nested in Habitat, with four sites per habitat type. Similarity matrices were calculated based on Bray–Curtis distances on square-root transformed data, which takes into consideration OTU abundances and identities ('community structure'), as well as on the Jaccard index, with focuses on OTU identities only (presence/absence data; 'community composition'). Analyses were done using 9999 permutations of residuals under a reduced model.^69^ PERMDISP was used to test for homogeneity of multivariate dispersion within groups.^70^ Non-metric multidimensional scaling (nMDS) was generated as an ordination method to visualise the variation of microbial community structure and composition in different sample groups.

GLMs were conducted to compare the relative abundances of each OTU among the three habitat types while considering the effect of sites using the R package ‘mvabund’ and assuming a negative binomial distribution.^71^ Because mvabund cannot deal with random effects, Site was fitted as a fixed effect in the model before habitat type to account for site variation, and tests used Type I (sequential) sums of squares. The relative abundance of over 200 OTUs differed significantly between habitats (Table S1), so for simplicity, further interpretation was limited to those OTUS where effects were strongest, defined here as those with an effect size larger than two times the standard error. A linear mixed-effects model was used to compare maximum quantum yield of kelp between the three habitat types, with Site fitted as a random effect in the model, in the R package lme4.^72^

### Data availability

Data generated and analysed during this study are included in this article’s supplementary information. Raw sequences are available through the Sequence Read Archive, NCBI (Submission ID: SUB3258310, BioProject ID: PRJNA419831).

## Electronic supplementary material

Table S2

Table S1

Figure S1
