# Supplementary figures and images for: Coastal urbanisation affects microbial communities on a dominant marine holobiont
Source: NPJ Biofilms Microbiomes. 2018 Jan 17;4:1. doi: 10.1038/s41522-017-0044-z (PMC5772048; doi:10.1038/s41522-017-0044-z)

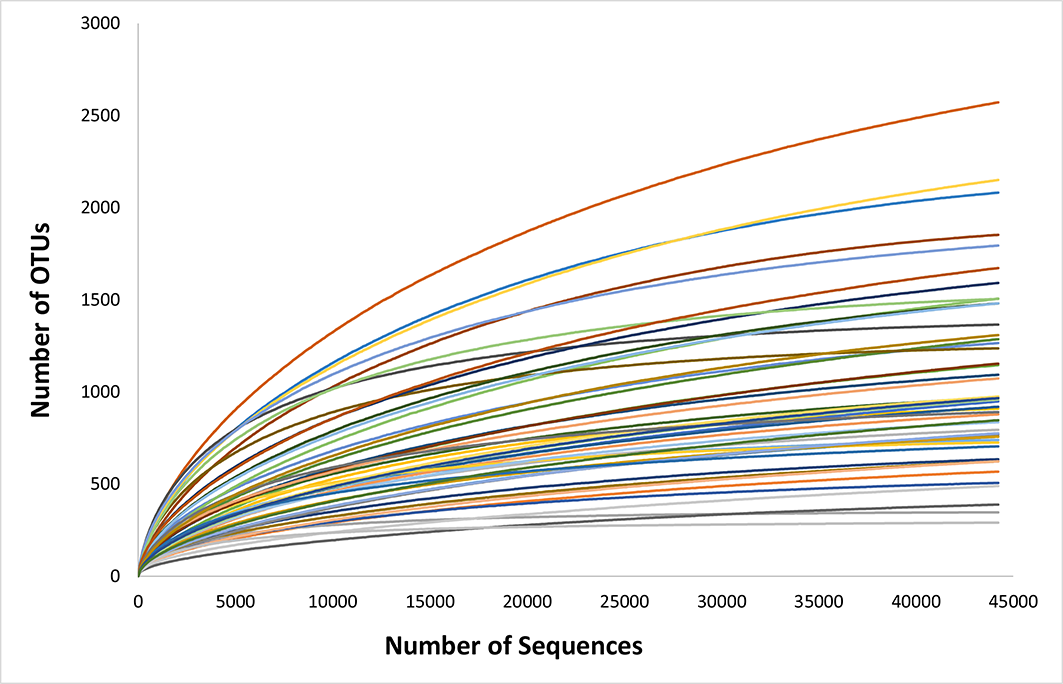

Supplement: Supplementary file 3 — Figure S1 [file 41522_2017_44_MOESM3_ESM.tif]
